# On the developmental origin of intrinsic honesty

**DOI:** 10.1371/journal.pone.0238241

**Published:** 2020-09-10

**Authors:** Tai-Sen He, Lili Qin

**Affiliations:** 1 Economics Programme, School of Social Sciences, Nanyang Technological University, Singapore, Singapore; 2 Department of Psychology, National University of Singapore, Singapore, Singapore; University of Queensland, AUSTRALIA

## Abstract

Contrary to the self-interestedness assumption, numerous economic studies have documented that people are intrinsically honest. However, little is known about this trait’s developmental origin. This study examines whether and the extent to which children in early childhood incur the intrinsic lying cost. We modified the commonly used coin-flip task into a child-friendly ball-drawing task with 10 trials and conducted the experiment with 225 child participants aged three to eight years old. We found that—although young children, on average, told two lies in the task (an average winning rate of 71%)—they lied significantly less than the maximum level (i.e., lying 100% of the time). The pattern was largely similar across gender and the age range studied. Furthermore, our child subjects’ propensity to lie dropped by approximately 9% when they were randomly assigned to the treatment condition with an increased “perceived” intrinsic cost of lying. Overall, our results align with the innate morality hypothesis: young children, as young as three years old, are willing to give up pecuniary rewards in order to remain honest.

*“The moral sense*, *or conscience is as much a part of man as his leg or arm.”*

—Thomas Jefferson (1787)

## I. Introduction

A unique trait differentiating human beings from other animals is the capacity to engage in extremely large-scale cooperation with unrelated humans. A prominent example is the global economic system, which coordinates virtually every single person in the world to produce goods and services to meet global human needs. Although self-interest and its corresponding incentive-compatible schemes ensure the smooth functioning of the system, trust is essentially the foundation of the human economic system. In the absence of adequate trust, people can barely engage in transactions—how many people would dare to deposit their money in banks or invest in stock markets if they did not trust the institutions [1]? Would people believe in “organic” food? Would anyone walk into a for-profit hospital for medical treatments? Would parents have any peace of mind while their young children were in a private childcare facility? Ultimately, how many market institutions could survive without people sufficiently believing that others would “do the right thing” even when it was not perfectly aligned with their own self-interest?

Importantly, then, economists must understand why the vast majority of people choose to behave honestly and morally. Tracing back to the 1980s, [2] proposed a novel theoretical framework arguing that morality (e.g., blushing when lying) is an evolved human trait as it functions as a commitment device to enhance people’s propensity to cooperate even if the ensuing action deviates from the Nash equilibrium. More recently, accumulating empirical evidence has demonstrated that people are intrinsically honest: even in a tightly controlled condition in which subjects have a clear monetary incentive to make a false statement, which can neither be detected nor affect others’ payoffs, they still lie very little [3–10].

Despite the prevalence of intrinsic honesty among the adult population and its importance in sustaining the exchange-based economy, very little attention has been paid to understanding the developmental origin of this behavioral inclination. Do people acquire it solely from social and cultural learning? Or is it an innate predisposition? Understanding the root cause of intrinsic honesty may influence how we think about the evolutionary basis of the human economic system.

This fundamental question can be partially addressed by examining whether young children, especially younger ones, also incur the intrinsic lying cost as they are less influenced than older children and adults by cultural and environmental factors [7]. For this purpose, we performed an incentive-compatible test of intrinsic honesty on the youngest children studied to date. More specifically, we modified the popular coin-flip paradigm into a young-child-friendly version and implemented it on a total of 225 child subjects aged three to eight years. In each of the 10 trials, participants privately drew a ball from an opaque box containing equal numbers of red and blue balls and were asked to report the ball color to the experimenter. If a red ball was reported, the child could choose a reward from a box of mixed tokens—which included candies, biscuits, and stickers—while reporting a blue ball yielded no reward. Obviously, child participants could win rewards by misreporting the outcome of the private ball-drawing without being detected.

Moral and developmental psychology has a long history of exploring children’s moral judgment and behaviors. However, unlike economists who analyze human behaviors from the self-interest perspective, psychological research in this area has yet to directly examine the reasons people, including young children, do not lie maximally. In fact, intrinsic honesty or intrinsic lying cost is rarely discussed in the psychological literature. As a result, although whether human morality is innate has been a decades-long question in several related fields—such as psychology, anthropology, biology, and even philosophy—and increasingly more recent evidence suggests that people are born with initial moral senses [11–14], none of the existing studies thus far serves as a satisfactory test of intrinsic honesty by meeting the strict conditions required by economists.

The test of intrinsic honesty should meet four conditions: 1) subjects have a clear pecuniary incentive to lie; 2) lying cannot be detected; 3) lying does not affect others’ payoffs; and 4) experimenters provide no hint of what is right or wrong behavior. To our best knowledge, the existing psychological experiments on children’s lying violate at least one of the above conditions. For example, the most frequently used task by psychologists studying children’s lying behavior is the temptation resistance paradigm developed by [15]. In this paradigm, a child participant is typically told explicitly by a researcher not to peek at or play with a toy when left alone and then is asked if he or she peeked when the researcher returns. This paradigm violates both conditions 2) and 4) as subjects are told that peeking at the toys is a wrong behavior and that their lies can be subsequently detected, either through a follow-up question or via video recordings from a hidden camera. On the other hand, in several important psychological research studies examining very young children’s moral senses—including [11, 16]—subjects simply indicated their preference without making an “incentivized” decision, which thus violates condition 1). Even for several psychological works that involve pecuniary rewards to incentivize subjects’ lying (e.g. hide and seek paradigm), conditions 2) and/or 3) are typically violated as subjects’ lies can be subsequently detected and/or have impacts on others’ payoffs.

On the other hand, although the economic literature on intrinsic honesty is fast-growing, very few attempts were devoted to exploring the lying behaviors among children, with the exception of [17, 18], and [19]. Although they all found that child subjects, like adults, also incurred the intrinsic cost of lying, these studies focused on children in middle childhood and/or adolescents. Therefore, there remains no thorough understanding regarding the ontogenic development of the intrinsic lying cost in early childhood.

To fill the gap in the psychological literature on innate morality and the economics literature on intrinsic honesty, the present study aims to perform possibly the strongest test of intrinsic honesty on the youngest children studied to date. Put simply, we test whether young children, as young as three years old, are willing to give up pecuniary rewards in order to remain honest. Another important feature distinguishing our study from prior research on intrinsic honesty is that we focus on non-WEIRD samples—participants from non-Western, educated, industrialized, rich, and democratic societies—and thus, the results extend our understanding of the universality of this behavioral tendency.

Our main findings are as follows. First, although the experimenter cannot verify the outcome for each ball draw, the extent of lying can be measured by the difference between the reported distribution of red balls across the two conditions to the distributions of fair draw (50% red balls reported) and profit maximization (100% red balls reported). We found that, while the child subjects told an average of two lies in the form of misreporting the ball color (an average winning rate of 71%), they clearly lied substantially less than the maximum level of 100%. Notably, we did not find any significant age effect, meaning that the intrinsic cost of lying emerges from very early stages of life, and this pattern remains similar across the ages of three to eight years old. Second, our subjects’ propensity to lie dropped by 9% when they were randomly assigned to the treatment condition with a heightened “perceived” intrinsic cost of lying. Overall, these findings constitute evidence that children in early childhood, like adults, also incur the intrinsic cost of lying.

## II. Experimental design and procedures

### 2.1 Procedures

We recruited child participants aged three to eight years old by advertising with local parenting groups and by liaising with childcare centers. The majority (80.4%) of the parents who brought their children in were mothers as they are often the main caregiver of children. All sessions, which each took approximately a half-hour to complete, were conducted in a small multifunctional seminar room at a major research university in Singapore (see Appendix A in S1 Appendix for more detail on the lab setting). Instructions were given verbally by female research assistants (see Appendix B in S1 Appendix for experimental instructions). All study participants provided informed consent and the study design was approved by Nanyang Technological University Institutional Review Board (IRB-2018-01-045).

The experiment proceeded as follows. Verbal and written consent were obtained from parents and children before the study. Then, experimenters demonstrated and conducted the ball-drawing task, a modified version of the coin-flip paradigm. The ball-drawing task required child participants to randomly draw a ball from an opaque box containing equal numbers of red and blue balls. The child would report the result of his or her ball draw to an experimenter, whose view was blocked by a black screen to eliminate concerns about lie detection. If a red ball was reported, the child could choose a reward from a box of mixed tokens. However, if a blue ball was reported, no reward was given. The task consisted of ten trials for each child. We did not explicitly tell the subjects they could misreport the observed ball color to earn tokens.

A training phase was conducted to ensure that each child participant fully comprehended the task and to overcome potential learning effects. More specifically, experimenters checked whether the child subjects understood that they could receive a reward only if a red ball was reported and that they knew how to report the draw outcomes according to the treatment conditions. If a child failed the comprehension check, the training phase was repeated. Before the real ball-drawing task began, the child was asked two questions: “Can you see me?” and “Can you see Mummy/Daddy?” The child was then reassured that neither his/her parent nor the experimenter was able to see the child’s action, eliminating concerns about lie detection. The child proceeded to complete ten successive ball draws with replacement (i.e. the drawn ball was placed back into the box for the next draw). At the end of the experiment, the child’s parents completed a post-experiment questionnaire on their demographic characteristics.

### 2.2 Linguistic Manipulation

We complemented our design with a linguistic manipulation that aimed to heighten the “perceived” intrinsic lying cost. If subjects incur the intrinsic cost of lying, they are predicted to reduce the propensity to lie in the treatment condition with a higher perceived lying cost. We designed two treatment conditions in which unobtrusive manipulation was naturally embedded into the ball-drawing task on a trial-by-trial basis. If child subjects were randomly assigned to the heighted perceived lying cost (HC) condition, they were instructed to report the outcome of the ball-drawing using a first-person pronoun by saying “*I got a red/blue ball*.” Otherwise, they simply said “*red/blue ball*” in the control condition.

Our linguistic manipulation was motivated by the existing studies in psychology and communications that have previously demonstrated that individuals use fewer self-oriented pronouns (e.g., “I” and “me”) when lying than when telling the truth, possibly to distance themselves from the lies [20, 21]. The psychological literature on self-awareness also supports our hypothesis. Self-awareness refers to individuals’ capacity to take themselves as the object of thought—people can think, act, and experience, and they can also think about what they “themselves” are thinking, doing, and experiencing. Children typically demonstrate self-awareness by passing the Rouge test before they reach the age of two. According to [22] objective self-awareness theory, inducing self-awareness can increase one’s self-evaluation and enhance sensitivity to social and moral norms, rules, and standards. Numerous studies in psychology provide extensive support for this theory with evidence from adults and children. For example, [23] reveal that children are more likely to adhere to the rule (taking only one candy from the bowl) when they are individuated (by being asked their names and where they live) and a mirror is placed in front of the candy bowl to induce their self-awareness. In engaging with lying behavior, individuals with greater self-awareness are more likely to evaluate their lying behavior with internal standards and thus come into greater conflict with moral values. Consequently, the mandatory use of the pronoun “I” heightens the “perceived” intrinsic lying cost and is predicted to reduce children’s propensity to lie.

## III. Results

### 3.1 Participants

A total of 240 children were recruited and participated in the experiment. Data from fifteen subjects was excluded, mainly due to the child subjects’ inability to adhere to the instructions given by experimenters, such as refusing to make any decisions, simply staying silent, and needing their parents to sit next to them. Thus, data from 225 child participants was valid for analysis. The age of the children ranged from three to eight years, with an average of five (s.e. = 1.3) years. Half (49.8%) of the participants were girls. Additionally, 91.1% of the participants were Chinese, and nearly all (99.1%) were Singaporean citizens or permanent residents. Table 1 provides summary statistics of the participants by treatment. There were no statistical differences for all of the demographic variables between the two treatment conditions except for a marginally significant difference in the age variable, which will be included as a control in regressions.

**Table 1:** Summary statistics.

Treatment	Control	HC Treatment	All Subjects
Female	0.5088	0.4865	0.4978
	(0.5021)	(0.5020)	(0.5011)
Age	4.8772	5.2072	5.0400
	(1.2701)	(1.3220)	(1.3036)
Chinese	0.9035	0.9189	0.9111
	(0.2966)	(0.2742)	(0.2852)
Singapore Citizen/PR	0.9825	1.0000	0.9911
	(0.1319)	(0.0000)	(0.0941)
No. of Observations	114	111	225

Standard deviations are reported in parentheses. Proportion tests (in the case of binary variables) and t-tests comparing demographics by treatment do not show statistically significant differences except for a marginally significant difference in age. These demographic variables are controlled in regression analysis.

### 3.2 Aggregate results

**Fig 1:**
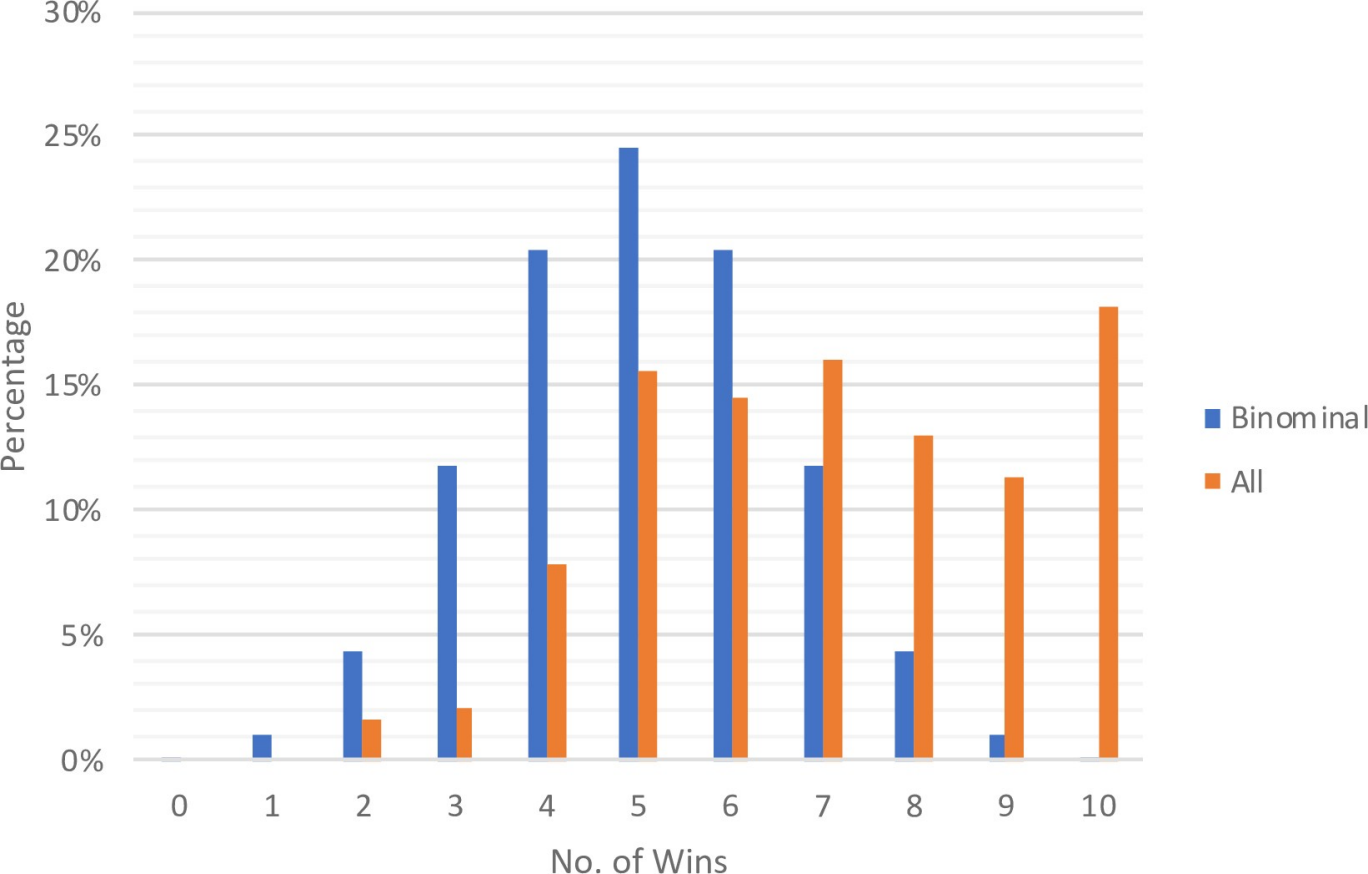
Distribution of wins reported by child subjects. Distribution of wins reported by subjects in both control and treatment conditions in comparison to the binomial distribution implied by honest reporting.

In line with other studies in the economic literature, we cannot verify participants’ answers to directly observe lying for each individual trial. Instead, we compared the distribution of reported wins (red balls) to that of the expected rate to infer whether and the extent to which lying occurred. Across all treatments, our sample child subjects reported an average of seven wins (red balls), indicating that they told an average of two lies when reporting the ball color throughout the ten trials. The success rate was significantly higher than 50% (p < 0.01, two-sided one sample t-test) and significantly lower than 100% (p < 0.01, two-sided one sample t-test). The overall distribution of reported red balls for the total sample of 225 children is shown in Fig 1. Not surprisingly, the distribution is right-skewed as child subjects tended to over-report the number of red balls; however, most of them lied partially, and in fact, less than one-fifth (18.7%) of the subjects lied to the maximum level (i.e., 100% of the time) by reporting a total of ten red balls. These results suggest that, although young children tell lies by misreporting the ball color to increase their pecuniary benefit, they are likely to incur the intrinsic cost of lying. Hence, the average winning rate was substantially lower than 100%.

**Result 1**: **The average number of wins was significantly lower than the maximum level of ten wins, suggesting that children in early childhood, like adults, incur the intrinsic cost of lying.**

Next, we considered the impact of gender and age on lying behavior. Gender differences in ethical preferences among adult populations have been well-documented in previous studies [24], but the results are rather ambiguous among child subjects. For instance, [18] did not note any gender difference, while [19] show that this gender gap disappears among older child subjects. In our study, we did not find a significant gender difference in lying among our sample child subjects across the two treatment conditions (p > 0.1, two-sided t-test). Regarding the age effect, we followed [25] and categorized our child subjects into three age groups: 3/4 (n = 84), 5/6 (n = 111), and 7/8 (n = 30). The average number of wins is 7.25 in the 3/4 group, 6.99 in the 5/6 group, and 7.10 in the 7/8 group, respectively. Again, we did not find any significant difference in lying among any two of the three age groups (p > 0.1, two-sided t-test).

**Result 2**: **The pattern of intrinsic lying cost was similar across gender and the age range from three to eight years old**.

### 3.3 The heightened perceived lying cost treatment effect

**Fig 2:**
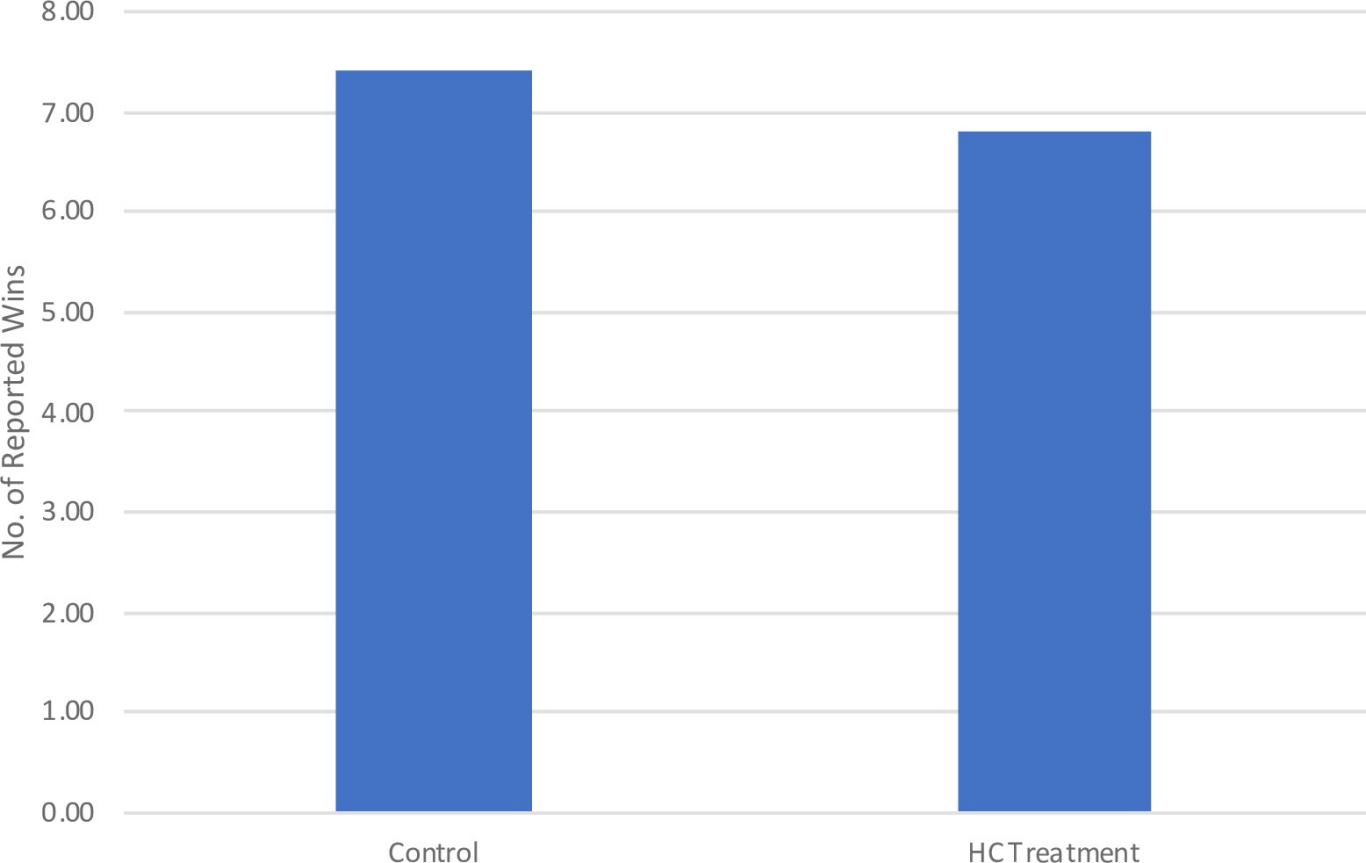
The impact of heightened perceived lying cost treatment on dishonesty. This figure illustrates the treatment effect of the linguistic manipulation that aimed to heighten the perceived intrinsic lying cost. The child subjects in the HC treatment condition reported an average of 6.8 wins, which is significantly lower than the that of the control group (P = 0.03, two-sided Mann-Whitney test; n = 225).

**Fig 3:**
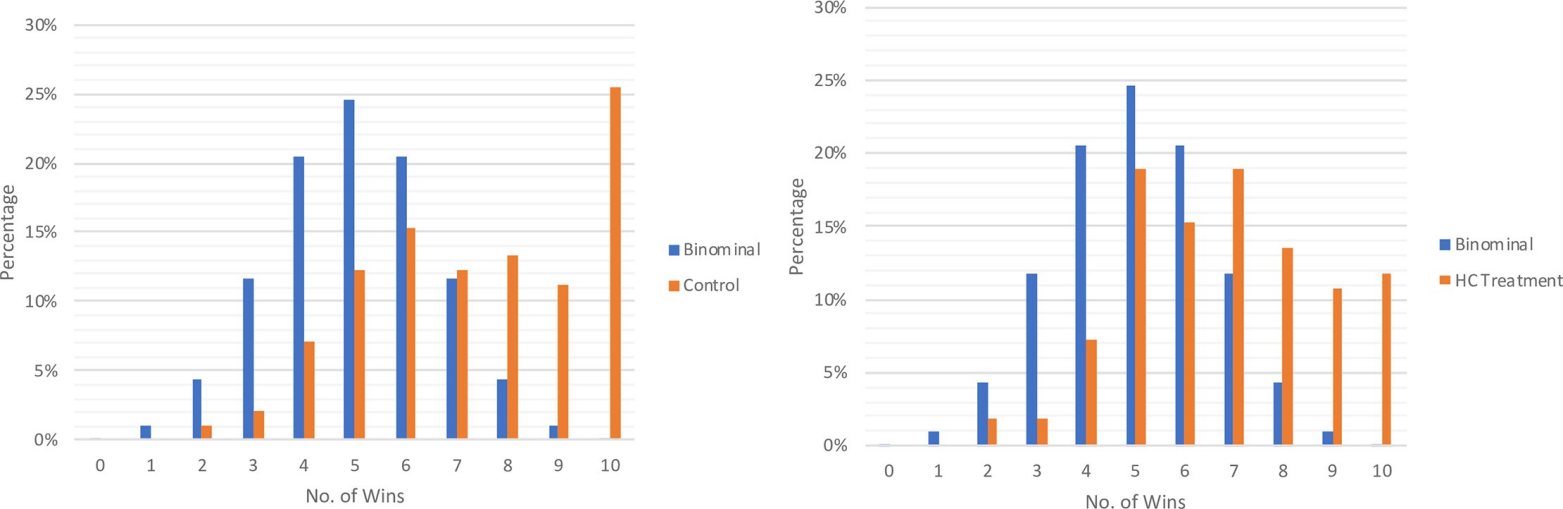
Comparison of the distribution of wins reported by treatment. (A) Distribution of reported wins in the control condition in comparison to the binomial distribution implied by honest reporting. (B) Distribution of reported wins in the HC treatment condition in comparison to the binomial distribution.

We predicted that, if our sample child subjects incurred the intrinsic cost of lying, the mandatory use of the pronoun “I” in reporting ball color would heighten the perceived intrinsic lying cost and thus reduce their propensity to lie, compared to the control condition. Fig 2 illustrates the treatment effect. Consistent with our prediction, those in the HC group reported an average of 6.8 wins, while those in the control group reported an average of 7.4 wins (p = 0.03 using two-sided Mann-Whitney test). Such outcomes mean that the subtle linguistic manipulation reduced the winning rate by roughly 9% (or six percentage points). In addition, we compared the distributions of reported red balls in the HC treatment condition vs. the control condition, as shown in Fig 3. The difference was statistically significant at 5% using the Kruskal-Wallis equality-of-populations rank test. Notably, the proportion of complete liars dropped largely from 25.4% in the control condition to 11.7% in the HC condition. These results reveal the effectiveness of using the first-person pronoun “I” in reducing young children’s propensity to lie. Importantly, the fact that young children respond positively to this manipulation provides another piece of evidence that they incur intrinsic or moral costs when telling lies.

**Result 3**: **The heighted perceived lying cost through mandatory use of the pronoun “I” significantly reduced young children’s propensity to lie**.

### 3.4 Probit regression analysis

The probit regression reported in Table 2 provides additional evidence for Results 1–3. Here, the dependent variable is reporting a win (1 for red ball and 0 otherwise). First, we regressed on the treatment dummy only and observe lower frequency of lying in the HC condition (coefficient of -0.06, P = 0.02), as illustrated in Column 1. Column 2 shows the results from exploring the period effect. The coefficient of *Period* is significantly positive, suggesting the learning effect. Perhaps child participants better understand the material benefits of lying throughout the experiment and therefore are more likely to lie with periods. Alternatively, once a child reports a red ball and receives a token, he or she may become more tempted to receive more tokens and thus lie more often. Even more, the subjects could potentially adapt themselves to lying with repetition [26]. Despite this, the winning rate of 76.9% in the last trial remained substantially lower than the maximum level of 100%. We further controlled for demographic variables, including gender (Female = 1 if the child subject was a girl; otherwise, Female = 0), age (the child subject’s age in years), race (Chinese = 1 if the parent reported the child subject’s race to be Chinese; otherwise, Chinese = 0), and immigration status (Citizen or PR = 1 if the parent reported the child’s immigration status to be Singapore citizen or permanent resident; otherwise, Citizen or PR = 0), as shown in Column 3. None of the coefficients of these demographic controls were statistically significant, meaning that the intrinsic lying cost was not associated with these demographic characteristics, corroborating Result 2.

**Table 2:** Effect of heighted lying cost on dishonesty.

	(1)	(2)	(3)
Dependent Variable:	1 if Successful Outcome		
HC Treatment	-0.0611**	-0.0610**	-0.0610**
	(0.0273)	(0.0273)	(0.0283)
Period		0.0072**	0.0075**
		(0.0029)	(0.0030)
Female			0.0363
			(0.0265)
Age			-0.0089
			(0.0104)
Chinese			-0.0065
			(0.0423)
Citizen or PR			0.1260
			(0.1211)
Observations	2250	2250	2250
No. of clusters	225	225	225

Probit estimates. Reported results are average marginal effects. Robust standard errors corrected for clustering on the individual level are in parentheses. The decision to report a win is regressed on a dummy for the HC treatment condition in Column (1). Column (2) includes period effect. Column (3) further controls for individual characteristics.

*** Significant at the 1% level.

** Significant at the 5% level.

* Significant at the 10% level.

## VI. Conclusion

Although several recent economic studies have documented that adults incur a high intrinsic cost of lying, very limited research has examined whether and the extent to which children, especially very young ones, incur the intrinsic lying cost. Using an economics approach, this study revealed the prevalence of the intrinsic lying cost among young children between 3 and 8 years old. Furthermore, child subjects’ propensity to lie was significantly reduced when they were randomly assigned to the treatment condition where subjects are required to report ball color using the pronoun “I” to aggravate the “perceived” intrinsic lying cost. Overall, our results demonstrate that the intrinsic lying cost emerges at very early stages of one’s life, and its pattern remains similar across the age range between 3 and 8 years old. We suggest that future research should systematically explore the prevalence of the intrinsic cost of lying in separate developmental stages to gain a fuller understanding of the progressive trajectory of the intrinsic lying cost.

In sum, to our best knowledge, this is the first study in economics and related fields that performs the test of intrinsic honesty on the youngest children studied to date. Our results shed important light on the developmental origin of intrinsic honesty and provide further support in line with the innate morality hypothesis from an economics perspective. We acknowledge that, while three- to four-year-old children are relatively young, we still cannot fully rule out the possibility that this behavioral inclination has been (at least partly) culturally learned and internalized before the age of three. As such, we cannot decisively conclude that humans are born to be intrinsically honest. Future research should consider performing the test on children at an even younger age. Nonetheless, our study makes an important contribution to the literature by revealing that human beings as young as three years old have started to exhibit intrinsic honesty in an economically meaningful way—they are willing to give up pecuniary rewards in order to remain honest.

## Supporting information

S1 Data (XLSX)

S1 Appendix (DOCX)
