# Supplementary material for: On the developmental origin of intrinsic honesty
Source: PLoS One. 2020 Sep 10;15(9):e0238241. doi: 10.1371/journal.pone.0238241 (PMC7482926; doi:10.1371/journal.pone.0238241)
Supplement: S1 Appendix — (DOCX) [file pone.0238241.s002.docx]

**Appendix A: Lab Setup**

1. Experimenter and child subject sit on different sides of the black screen.


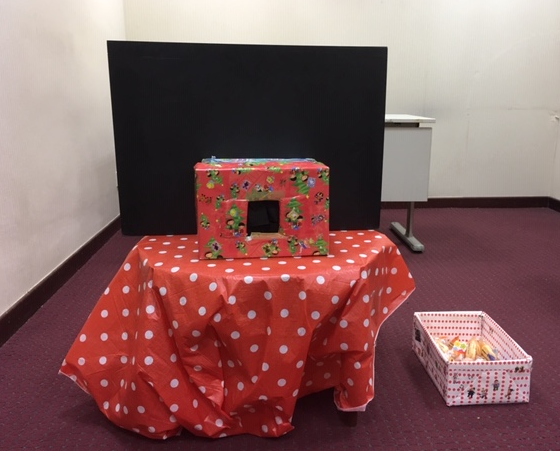


1. A black cloth is attached to the opening of the box to prevent children from seeing into the box and “finding” a red ball.


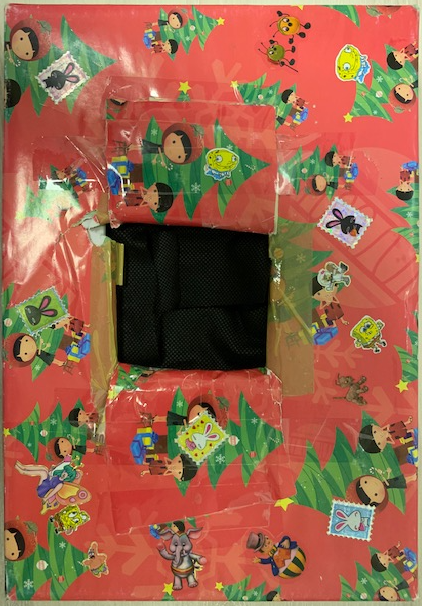


1. Inside of the ball-drawing box, there are six red and six blue balls.


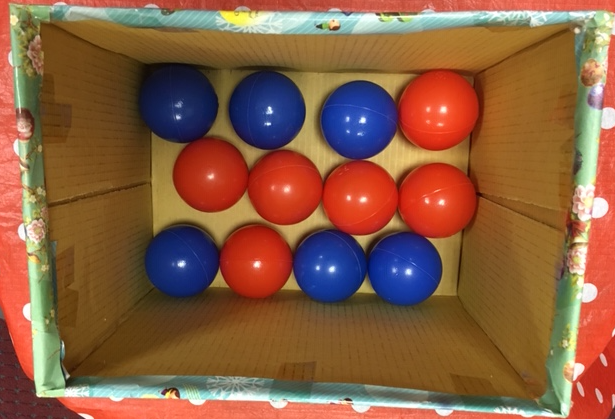


1. Tokens includes candies, biscuits, and stickers.

**
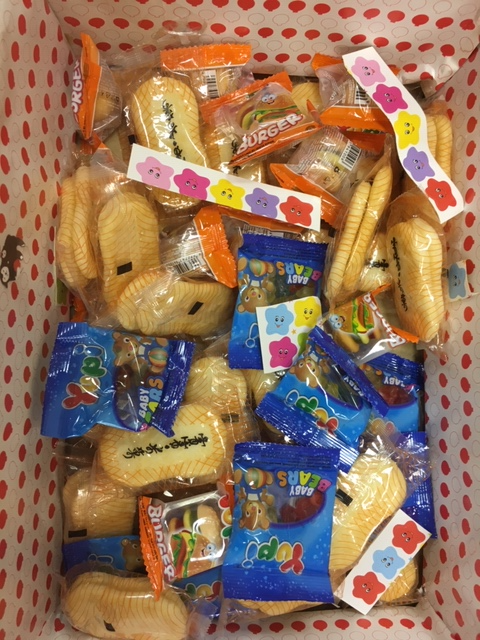
**

**Appendix B: Experimental Instructions for the Ball-Drawing Task**

**Pre-task Parent Briefing**

Give briefing on procedure and to refrain from giving comments to the child.

1. **Training Phase:**
2. ***Experimenter demonstration***

| **SAY** | **DO** |
| --- | --- |
| **Briefing by Experimenter 1**  “Today we will play a fun game. Let me and (E2) show you how to play ok? Now sit together with (E2) and learn how to play the game”.  E2 will take a ball from this box, tell me the colour of the ball, and put it back into the box. There are two different coloured balls in the box: red and blue.  If E2 gets a red ball, E2 can choose 1 thing from this box (reward)  If E2 gets a blue ball, she would get nothing.  We show you how to play ok? | E1 sits **behind** screen.  E2 sits with child on the same side. |
| **Red Ball**  Experimenter 1: “Take a ball”  Experimenter 2: **“(I got a) red ball”**  Experimenter 1: Ok now put the ball back into the box.  Experimenter 1: “Yay! Choose a present from the box.”  Experimenter 2: (Explain to the child) “I need to say **“(I got a) red ball”**. **(depending on the treatment condition)** Because the ball is red, I can take 1 present.” | E2 draws ball, shows child  E2 puts ball back  E2 takes reward |
| **Blue Ball**  Experimenter 1: “Take a ball”  Experimenter 2: **“(I got a) blue ball”**  Experimenter 1: Now put the ball back into the box.  Experimenter 1: “Sorry, you get nothing.”  Experimenter 2: (Explain to the child) “I need to say **“(I got a) blue ball”**. Because the ball is blue, I cannot take anything.” | E2 draws ball, shows child  E2 puts ball back |

1. ***Comprehension check***

| **SAY** | **DO** |
| --- | --- |
| Experimenter 1: “Take a ball”  **Experimenter 2: (ask child) “What should I say?”**   - **The child should report “(I got a) red/blue ball”** - Correct the child if the child merely says “red/blue”. | E2 draws ball  (Wait for answer) |
| Experimenter 1: “Put the ball back into the box.”  **Experimenter 2: (ask child) “What should I do now?”**   - **The child should say “put the ball back.”** - If correct – encourage child: “Yes, that’s correct. I put the ball back” - If wrong – remind child | (Wait for answer) |
| Experimenter 1:   - If red: “Yay! You can select a present from the box.” - If blue: “Sorry, you get nothing.”   **Experimenter 2: (ask child) “What should I do now?”**   - **Child should say take/not take a reward.** - If correct – Encourage the child:   - If Red: “Yes that’s correct. Because the colour is red, I can take 1 present”;   - If Blue: “Yes that’s correct. Because the colour is blue, I cannot take a present.” - If wrong – correct the child, show correct behaviour | (Wait for answer) |
| **Repeat step 2 until the child can succeed 3 times in a row.**  (i.e. Can correctly report colour + knows in what situation she/he could get a reward or not)   - If **pass**:   - Stop the training: “This is pretty fun, right? Do you want to play the game?”   - **Continue to Study Phase** - If **fail**: Repeat Training Phase | |

1. **Study Phase:**
2. ***Briefing***

| **SAY** | **DO** |
| --- | --- |
| Remind parents not to make any comments or actions during the full course of the Study Phase, except when prompted by the experimenter.  Ensure child is seated behind screen. | E2 leaves |
| **Parent View Check (done by E1)**  “You have learned how to play this game! Now I have a question for you.”  Q: “When you sit behind this screen, can you see mommy/daddy?”  Q: “Do you think mommy/daddy can see you?”  Q: Let’s check. “Mommy/daddy, can you see (child’s name)’s face?”   - Ensure parent responds verbally - If parent can see child, adjust accordingly.   “You cannot see mommy, mommy cannot see you. So, mommy doesn’t know what coloured ball you have. Only you know.” | Wait for child’s answer |
| **Experimenter View Check**  *Sit behind screen*  Q: “When you sit behind this screen, can you see me?”  Q: “Do you think I will know what ball you get or not?”   - Response: “That’s right/Nope, I cannot see you!”   “You cannot see me, I cannot see you. So, I don’t know what coloured ball you have. Only you know.” | Wait for child’s answer |
| **Reminder**   - If the colour is RED, you will GET a present - If the colour is BLUE, you will NOT get a present - You should say loudly **“I got a red/blue ball”** OR **‘Red Blue Ball’**. Okay? - Now we will start. |  |

1. ***Study Phase***

| **SAY** | **Remarks** |
| --- | --- |
| “Draw a ball”  *Child draws ball.*  Q: What did you get?  *Experimenter notes: Record reported colour*  If child does not follow condition, record response as null and remind child again.  “Ok, put the ball back into the box.” | Ensure child responds, **“I got a red/blue ball”** *OR* **“red/blue ball”**  Ensure child puts ball back. |
| If red: “Yay! Choose a present from the box”.  If blue: “Sorry, you get nothing”  Let’s try again! | Ensure child takes 1 reward only.  *shake box again |
| **Repeat steps for 10 times** |  |
